# Leiomyoma of the Vulva: A Diagnostic Challenge Case Report

**DOI:** 10.1155/2016/8780764

**Published:** 2016-10-12

**Authors:** Saad Kurdi, Abdullah S. Arafat, Maysan Almegbel, Mayson Aladham

**Affiliations:** ^1^King Abdulaziz Medical City (KAMC), Riyadh, Saudi Arabia; ^2^King Saud bin Abdulaziz University for Health Sciences (KSAU-HS), Riyadh, Saudi Arabia

## Abstract

Uterine leiomyomas are common and can affect up to 30% of women older than 35 years. Despite this, leiomyomas of the vulva are rare, masquerading, and usually misdiagnosed as Bartholin cyst preoperatively. These smooth muscle tumors are typically painless, solitary, and well circumscribed and can affect female of any age group. We present a case of a 46-year-old female that presented to the clinic with 2-year history of right labial mass and was diagnosed as Bartholin cyst initially. The patient underwent elective excision under spinal anaesthesia and the mass was removed. The final diagnosis after microscopy result showed benign vulvar leiomyoma.

## 1. Introduction

Leiomyoma of vulva is a rare, benign mass that is present on the vulva. Uterine leiomyomas are common and can affect up to 30% of women older than 35 years [1]. Despite this, leiomyomas of the vulva are masquerading. These smooth muscle tumors present a greater diagnostic challenge and are usually misdiagnosed as Bartholin cyst preoperatively. Less than 160 cases have been reported in the literature [1–3]. Vulvar leiomyomas are typically painless, solitary, and well circumscribed. They can affect female of any age group but mostly between 30 and 60 years of age. Histologically, vulvar leiomyomas originate from smooth muscle within erectile tissue, blood vessel walls, and the round ligament [4]. Typical vulvar leiomyomas demonstrate spindle shaped cells, but other histological types such as epithelioid tumors are also reported [5]. Here we report a case of 46-year-old lady with vulvar leiomyoma which was misdiagnosed as Bartholin cyst preoperatively.

## 2. Report of the Case

A 46-year-old female P4 +0 presented to the clinic with 2-year history of right labial mass. The mass showed mild progression over that period and was associated with pain. There was no history of discharge, fever, or weight loss. General examination was unremarkable except for a soft mass that measured 4 × 3 cm in the right labial area. The mass was medial to the right labia minora. The patient had a history of four spontaneous vaginal deliveries without induction. The family history was unremarkable.

The mass was diagnosed initially as a Bartholin cyst. She was counseled for the management options and agreed to undergo surgical intervention. She was booked for day care surgery. An elective excision under spinal anaesthesia was performed. The incision at the mucocutaneous junction showed soft, fleshy, and well defined mass measuring 3 × 4 cm. The mass was enucleated in fragments and sent to the histopathology lab. The patient had a good recovery postoperatively with no complications.

**Figure 1:**
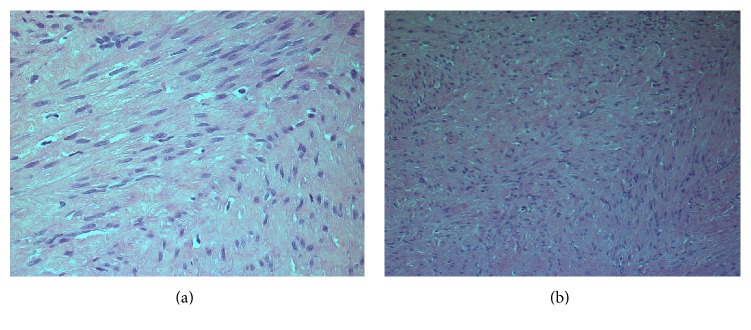
(a), (b) Microscopy showing a benign tumor composed of sheets and fascicles of oval to spindle shaped cells with abundant dense cytoplasm and areas of hyalinization.

On histopathological examination, the gross specimen consisted of two soft pieces of tissue measuring 4.1 × 2.3 × 1.8 cm. Slicing of the specimen revealed fleshy solid cut surface and no cyst was seen. Microscopy revealed benign tumor composed of sheets and fascicles of oval to spindle shaped cells with abundant dense cytoplasm and areas of hyalinization (Figures 1(a) and 1(b)). The final diagnosis was benign vulvar leiomyoma.

## 3. Discussion

Leiomyomas are benign tumors that arise from smooth muscle cells in possibly any anatomical site within the body [6]. Vulvar leiomyoma is a rare type since about 160 cases have been reported in English literature so far [1–3]. It has a number of histological origins including smooth muscle cells, spindle cells, and epithelioid cancer cells of eosinophilic cytoplasm [3]. When immunohistochemical stains are done, vulvar leiomyomas stain positive for estrogen receptors or progesterone receptors and sometimes both. Thus, treatment with receptor modulators in adjuvant to surgery may be beneficial [3, 7].

A common mistaken initial diagnosis for vulvar leiomyoma is Bartholin's cyst, since both share some of the presenting symptoms such as painless lump, redness, and swelling of the area. Some features that support the diagnosis of Bartholin's cyst are everted labia minora and cystic consistency of the swelling; however, finding inverted labia minora and firm consistency of the swelling support the diagnosis of vulvar leiomyoma [8].

In cases with vulvar leiomyoma, differentiating between benign and malignant lesions is somewhat challenging. Nielsen et al. proposed a criterion to distinguish between the two lesions based on 4 features: more than 5 cm in widest dimension, infiltrative margins, more than 5 mitotic figures per 10 hpf, and moderate to severe cytologic atypia. If 3 or all features were found, then the neoplasm is considered to be a sarcoma. Benign but atypical leiomyomas fulfill only 2 characteristics, and benign leiomyomas are the ones that exhibit 1 or none of the traits [7]. In reference to their perception, our case did not have any of the features mentioned above, which suggested the diagnosis of benign leiomyoma. Another method that may help in distinguishing between malignant and benign tumors is using MRI; unlike normal smooth muscle cells, a malignant growth shows low intensity signal on T2-weighted scans [1, 8].

Excision of the tumor with some of the surrounding normal tissue is the treatment of choice, for it decreases the rate of recurrence and increases the 5-year survival rate [7]. Radiotherapy and chemotherapy were sometimes used in high-grade tumors and in cases of recurrence of the disease [9].

## 4. Conclusion

Vulvar leiomyoma is a rare tumor that is commonly misdiagnosed as Bartholin's cyst. Distinguishing between benign and malignant forms is also confusing, which makes vulvar leiomyoma a great diagnostic challenge. As there are a few techniques used to differentiate between the natures of the tumor, excisional biopsy seems to be the best current method used in addition to being the treatment of choice for such tumors. Continuing follow-up after treatment is highly recommended.

## References

[1] Fasih N., Shanbhogue A. K. P., Macdonald D. B. (2008). Leiomyomas beyond the uterus: unusual locations, rare manifestations. *Radiographics*.

[2] Reyad M. M., Gazvani M. R., Khine M. M. (2006). A rare case of primary leiomyoma of the vulva. *Journal of Obstetrics and Gynaecology*.

[3] Zhao T., Liu X., Lu Y. (2015). Myxoid epithelial leiomyoma of the vulva: a case report and literature review. *Case Reports in Obstetrics and Gynecology*.

[4] Kaufman R. H., Gardner H. L. (1965). Benign mesodermal tumors. *Clinical Obstetrics and Gynecology*.

[5] Tavassoli F. A., Norris H. J. (1979). Smooth muscle tumors of the vulva. *Obstetrics and Gynecology*.

[6] Holst V. A., Junkins-Hopkins J. M., Elenitsas R. (2002). Cutaneous smooth muscle neoplasms: clinical features, histologic findings, and treatment options. *Journal of the American Academy of Dermatology*.

[7] Nielsen G. P., Rosenberg A. E., Koerner F. C., Young R. H., Scully R. E. (1996). Smooth-muscle tumors of the vulva: a clinicopathological study of 25 cases and review of the literature. *The American Journal of Surgical Pathology*.

[8] Pandey D., Shetty J., Saxena A., Srilatha P. S. (2014). Leiomyoma in vulva: a diagnostic dilemma. *Case Reports in Obstetrics and Gynecology*.

[9] Khosla D., Patel F. D., Kumar R., Gowda K. K., Nijhawan R., Sharma S. C. (2014). Leiomyosarcoma of the vagina: a rare entity with comprehensive review of the literature. *International Journal of Applied and Basic Medical Research*.

